# High-Throughput Metabolomics Platform for the Rapid Data-Driven Development of Novel Additive Solutions for Blood Storage

**DOI:** 10.3389/fphys.2022.833242

**Published:** 2022-03-14

**Authors:** Travis Nemkov, Tatsuro Yoshida, Maria Nikulina, Angelo D’Alessandro

**Affiliations:** ^1^Omix Technologies Inc., Denver, CO, United States; ^2^Department of Biochemistry and Molecular Genetics, University of Colorado Denver – Anschutz Medical Campus, Aurora, CO, United States; ^3^Hemanext Inc., Lexington, MA, United States

**Keywords:** transfusion medicine, storage, red blood cell, metabolism, methionine, high-throughput screening

## Abstract

Red blood cell transfusion is a life-saving intervention, and storage is a logistic necessity to make ~110 million units available for transfusion every year worldwide. However, storage in the blood bank is associated with a progressive metabolic decline, which correlates with the accumulation of morphological lesions, increased intra- and extra-vascular hemolysis upon transfusion, and altered oxygen binding/off-loading kinetics. Prior to storage, red blood cells are suspended in nutrient formulations known as additive solutions to prolong cellular viability. Despite a thorough expansion of knowledge regarding red blood cell biology over the past few decades, only a single new additive solution has been approved by the Food and Drug Administration this century, owing in part to the limited capacity for development of novel formulations. As a proof of principle, we leveraged a novel high-throughput metabolomics technology as a platform for rapid data-driven development and screening of novel additive solutions for blood storage under both normoxic and hypoxic conditions. To this end, we obtained leukocyte-filtered red blood cells (RBCs) and stored them under normoxic or hypoxic conditions in 96 well plates (containing polyvinylchloride plasticized with diethylhexylphthalate to concentrations comparable to full size storage units) in the presence of an additive solution supplemented with six different compounds. To inform this data-driven strategy, we relied on previously identified metabolic markers of the RBC storage lesion that associates with measures of hemolysis and post-transfusion recovery, which are the FDA gold standards to predict stored blood quality, as well as and metabolic predictors of oxygen binding/off-loading parameters. Direct quantitation of these predictors of RBC storage quality were used here—along with detailed pathway analysis of central energy and redox metabolism—as a decision-making tool to screen novel additive formulations in a multiplexed fashion. Candidate supplements are shown here that boost-specific pathways. These metabolic effects are only in part dependent on the SO_2_ storage conditions. Through this platform, we anticipate testing thousands of novel additives and combinations thereof in the upcoming months.

## Introduction

After vaccination, blood transfusion is the most common in-hospital procedure (Pfuntner et al., 2013) and a critical life-saving intervention for 3.5–5 million Americans annually. This statement holds true even despite the decline in usage that started in 2014, a trend brought about by the introduction of restrictive transfusion regimens and improved patient blood management strategies (Goel et al., 2018). Red blood cell (RBC) storage in the blood bank is a critical procedure that makes it logistically feasible to collect and store ~110 millions of units of blood donated in 13,282 centers across 176 countries around the world every year (George, 2018).

Despite these advancements in RBC storage strategies, there is room for improvement in blood storage (Yoshida et al., 2019), as storage in the blood bank promotes the accumulation of a series of biochemical and morphological changes to RBCs that ultimately impact their energy and redox metabolism (Rogers et al., 2021), protein membrane integrity (e.g., band 3 fragmentation; Issaian et al., 2021), morphology (D’alessandro et al., 2012), functionality *in vitro* (e.g., decreased 2,3-diphosphoglycerate and oxygen off-loading capacity; Tsai et al., 2010; Donovan et al., 2021), in animal models *in vivo* (Hod et al., 2010), and clearance upon transfusion (Roussel et al., 2021). This “storage lesion”—as it is collectively referred to—has the potential to negatively impact transfusion outcomes (Yoshida et al., 2019).

Impaired energy and redox homeostasis in stored RBCs contributes to increased intra-(D’alessandro et al., 2021a) or extra-vascular (Rapido et al., 2017) hemolysis after transfusion, which in turn could (i) decrease the capacity to counteract systemic hypoxemia in transfusion recipients (Donovan et al., 2021); (ii) increase the risk of septic complications, when in presence of siderophilic bacteria (La Carpia et al., 2019); (iii) increase the risk of inflammatory complications, in part mediated by bioactive lipids, heme, and iron that accumulate in the bloodstream of the recipient (Howie et al., 2019). Over the past decade, a long series of retrospective studies (Silliman et al., 1997; Koch et al., 2008; Goel et al., 2016; Caram-Deelder et al., 2017) and small-scale, adequately powered, controlled prospective clinical trials (Rapido et al., 2017) suggested that storage duration may negatively impact transfusion outcomes, especially in some categories of recipients at risk. Clinical studies have shown that storage duration negatively impacts (~17% decline at storage day 42; Dumont and AuBuchon, 2008; Mays and Hess, 2017) RBC capacity to circulate after 24 h from transfusion in healthy autologous volunteers (D’alessandro et al., 2019b), a necessary though not sufficient requirement to ensure the proper function of transfused RBCs. This loss of potency may even be more marked in non-autologous, non-healthy recipients, such as in the case of sickle cell patients (Kozanoglu and Ozdogu, 2018), where a pro-inflammatory environment could promote erythrophagocytosis of transfused red cells.

Over the past few years, a series of randomized clinical trials on the age of blood reassured about the non-inferiority of current storage strategies when compared against the preferential transfusion of the freshest units available (Belpulsi et al., 2017). From these studies, it emerged those factors other than just the age of blood impact the quality of stored RBC units. Of note, similar conclusions came from post-transfusion recovery studies in 2008 (Dumont and AuBuchon, 2008), as well as from the Recipient Epidemiology and Donor Assessment study (REDS III). In the latter study, significant heterogeneity in hemolytic propensity was noted as a function of donor biology [e.g., sex, age, ethnicity (Kanias et al., 2017), and body mass index (Hazegh et al., 2021)], dietary, or other exposures (including drugs, caffeine, alcohol, or nicotine exposures; Nemkov et al., 2020), and first and foremost processing strategies—including storage additives (D’alessandro et al., 2019a). Altogether, these studies suggested that the chronological storage age (days since donation) and metabolic age of the unit are two different concepts, with the latter representing a more accurate indicator of the quality of the RBCs in the unit (D’alessandro et al., 2019c). As such, the development of novel storage additives aimed at improving the metabolic phenotypes of stored RBCs could contribute to significantly boosting transfusion efficacy.

Despite the need for improvement, only one new additive solution has been approved by the FDA in the last 30 years (Additive Solution 7—AS-7 or SOLX®, Hess, 2006 approved in 2013), though it was never commercialized due to financial limitations associated with high implementation costs. Alkaline additives (D’alessandro et al., 2018) have been proven to boost RBC metabolism and post-transfusion recoveries (Cancelas et al., 2015), though logistical issues have hampered their implementation owing to the caramelization of solutions with alkaline pH during the process of sterilization. Thus, only three FDA-approved additive solutions are used for RBC storage today in the United States (AS-3 or Nutricel® patented in 1983, AS-5 or Optisol® patented in 1983, and AS-1 or ADSOL® patented in 1988; Hess, 2006). In parallel, outside the United States, other additive solutions have been developed and implemented solution in Europe (saline-adenine glucose and mannitol—SAGM, which was introduced in 1981), and later adopted in Australia, New Zealand and, most recently, in Canada. However, the poorer end-of-storage quality of SAGM-stored RBCs relative to FDA-approved additive solution such as AS-3 has pushed most countries in Europe to shorten the shelf-life of packed RBCs to 35 days, despite decreased RBC supply resulting from this regulation. Other additives have been approved in Japan and Europe (e.g., MAP and PAGGSM), though they have not been yet approved by the FDA.

Following an alternative route, over the past 15 years a solid body of evidence has accumulated, documenting the beneficial impact of hypoxic storage on energy and redox metabolism of stored RBCs (Yoshida et al., 2008; Dumont et al., 2016; Reisz et al., 2016). Notably, hypoxic storage mitigates and, in some instances, completely abrogates the storage lesion to the RBC (Yoshida et al., 2017) by removing a key substrate for the generation of reactive oxygen species—*via* Fenton and Haber-Weiss reactions—while normalizing post-processing heterogeneity in Hb oxygen saturation across donors (Yoshida et al., 2017). Through a combination of state-of-the-art metabolomics and fluxomics experiments (Nemkov et al., 2017b), we recently noted that *ex vivo* hypoxic storage rewires RBC metabolism similarly to metabolic reprogramming under *in vivo* hypoxic conditions, both under physiological (e.g., high altitude; Sun et al., 2015; D’alessandro et al., 2016b; Liu et al., 2016) or pathological (e.g., hemorrhagic shock; Reisz et al., 2017) conditions. Despite the lack of mitochondria and other organelles and lack of *de novo* protein synthesis capacity, the mature erythrocyte has evolved to leverage metabolic reprogramming as a strategy to cope with systemic hypoxia and improve tissue oxygenation (Nemkov et al., 2018a). This mechanism is controlled *in vivo* by metabolic changes in plasma, such as for example the extracellular accumulation of adenosine that promotes signaling through receptor A2b–ADORA2b on the RBC (Liu et al., 2016), or uptake *via* a specific ENT1 transporter (Song et al., 2017). While such plasma metabolic changes are driven by distal organ metabolism (e.g., liver, endothelial system) after exposure *in vivo*, in the closed system of a blood bag this control is not possible *in vitro*. Thus, alternative additives have to be designed to maximize the metabolic benefits of hypoxic storage.

Previous generation omics approaches are limited by throughput in terms of cost and time. To make large clinical cohorts amenable to metabolomics testing, we developed high-throughput approaches that allow a combination of untargeted, semi-targeted, quantitative and tracing experiments for the analysis of hydrophilic or lipophilic compounds (Nemkov et al., 2019). These methods advanced our capacity to investigate plasma and organ-specific responses to acute or chronic hypoxia with a special focus on cancer metabolism (Jones et al., 2020), trauma/hemorrhagic shock (Williams et al., 2020), immuno-metabolism and inflammation (Thomas et al., 2020), mammalian hibernation (Rice et al., 2020), and pulmonary hypertension (Zhang et al., 2017). As a proof of principle, here we leverage this novel technology as a platform for the rapid data-driven development and screening of novel additive solutions for blood storage under normoxic or hypoxic conditions. To this end, we obtained leukocyte-filtered RBCs and stored them under normoxic or hypoxic conditions in 96 well plates in the presence of an additive solution supplemented with six different compounds. To inform this data-driven strategy, we relied on previously identified metabolic markers of the RBC storage lesion (Paglia et al., 2016a), that associate with measures of hemolysis and post-transfusion recovery (D’alessandro et al., 2020; Francis et al., 2020), which are the FDA gold standards to predict stored blood quality, as well as and metabolic predictors of oxygen binding/off-loading parameters. Direct quantitation of these predictors of RBC storage quality were used here—along with detailed pathway analysis of central energy and redox metabolism—as a decision-making tool to screen novel additive formulations in a multiplexed fashion.

## Materials and Methods

### Storage in Parent Unit vs. 96 Well Plates

Whole blood units were donated by 12 healthy donor volunteers in CP2D (Haemonetics, Boston, MA, United States) and suspended in AS-3 additive solution after leukofiltration and plasma removal. Two compatible RBC units are pooled then split into normoxic control (N) and hypoxic/hypocapnic (H) subunits. H subunit was processed by Hemanext ONE kit for 3 h at room temperature (Hemanext, Lexington, MA, United States) to reduce oxygen content by ~22% of the N (pO_2_ ~15 mmHg) and pCO2 < ~30 mmHg. Hemanext One is a commercial RBC Processing and Storage System that has received a CE Mark in 2021. A volume of 150 ml storage bag containing diethylhexylphtalate (DEHP) plasticizers (Fenwal 4R2001, Fresinius Kabi) mimic the exact composition of routine storage bags (see also Stefanoni et al., 2020) were used to store half of the units, while the other half was used to test storage in 96-well plate (GBO PP-Masterblock) format. Only H subunits were further processed inside a N2-filled glove box (O_2_ < 0.2%) as previously described (Reisz et al., 2016). For 96-well plate storage experiments, each well contained 1.2-2ml of RBCs in additive solution (average hematocrit 61.8 ± 1.1%), with the addition of polyvinylchloride plasticized with diethylhexylphthalate to concentrations comparable to those detected in full size storage units at the end of storage, as described in D’alessandro et al. (2016a). Sterility during storage was maintained by sealing the plate with Al or clear sealing film. Additionally for hypoxic 96 well-plates, they were stored inside oxygen barrier bag with oxygen/CO_2_ sorbent pack. Both N and H 96 well plates were stored in the presence of the following supplements:

- Untreated (original AS-3 formulation);
- Adenosine (10 μM—Sigma Aldrich, St. Louis, MO, United States)
- L-Glutamine (1 mM—Sigma Aldrich, St. Louis, MO, United States)
- Methionine (1 mM—Sigma Aldrich, St. Louis, MO, United States)
- N-acetylcysteine (1 mM—Sigma Aldrich, St. Louis, MO, United States)
- Taurine (1 mM—Sigma Aldrich, St. Louis, MO, United States)

The concentrations of these pilot supplements to AS-3 were chosen as 10x physiological levels for each metabolite, as per the Human Metabolome Database,^1^ to sustain that metabolic pathway throughout storage, with the exception of adenosine that was kept below 14 μM to avoid complications with induction of arrhythmia in the recipient, if the additive would ever make it to the clinics.

A plate was generated per each condition (*n* = 6), either under N or H (separate plate) per each different time point, resulting in longitudinal sampling at storage day 0, 7, 14, 21, 28, 35, and 42 (seven time points). A total of 588 samples were thus generated for high-throughput metabolomics screening.

### Glucose Isotope Tracing Analysis

In a separate storage experiment, seven whole blood units were processed as above and suspended in AS-3 containing 5 mM ^13^C_2_-Glucose (Sigma Aldrich, St. Louis, MO, United States). Units were then pooled and split into two identical 96 well plates each containing 10 technical replicates. Oxygen content was reduced as described above, and samples were taken at 3 and 6 weeks of storage and frozen at −80°C until analysis.

### Sample Processing and Metabolite Extraction

Automated liquid handlers were used to transfer volumes for sample processing and extraction (Opentrons system). A volume of 10 μl of RBCs was suspended in 95 μl of ice-cold methanol:acetonitrile:water (5:3:2, *v/v/v*) and vortexed at 4°C for 30 min prior to a 96-well plate-compatible positive pressure-assisted filtration of the extracts. Filtered extracts were stored at −20°C until analysis.

### Ultra-High-Pressure Liquid Chromatography-Mass Spectrometry Metabolomics

Analyses were performed using a Vanquish UHPLC coupled online to a Q Exactive mass spectrometer (Thermo Fisher, Bremen, Germany). Samples were analyzed using a high-throughput 1 min gradient, as generally described (Nemkov et al., 2017a, 2019; Reisz et al., 2019). Solvents were supplemented with 0.1% formic acid for positive mode runs and 10 mM ammonium acetate +0.1% ammonium hydroxide for negative mode runs. MS acquisition, data analysis and elaboration was performed as described (Nemkov et al., 2017a, 2019; Reisz et al., 2019). Data were analyzed using El-Maven (Agrawal et al., 2019), MetaboAnalyst 5.0 (Pang et al., 2021), and figures were created with GraphPad Prism 9 and Biorender.com.

## Results

### Storage of RBCs in 96 Well Plate Is Comparable to Storage in a Plastic Bag

**Figure 1:**
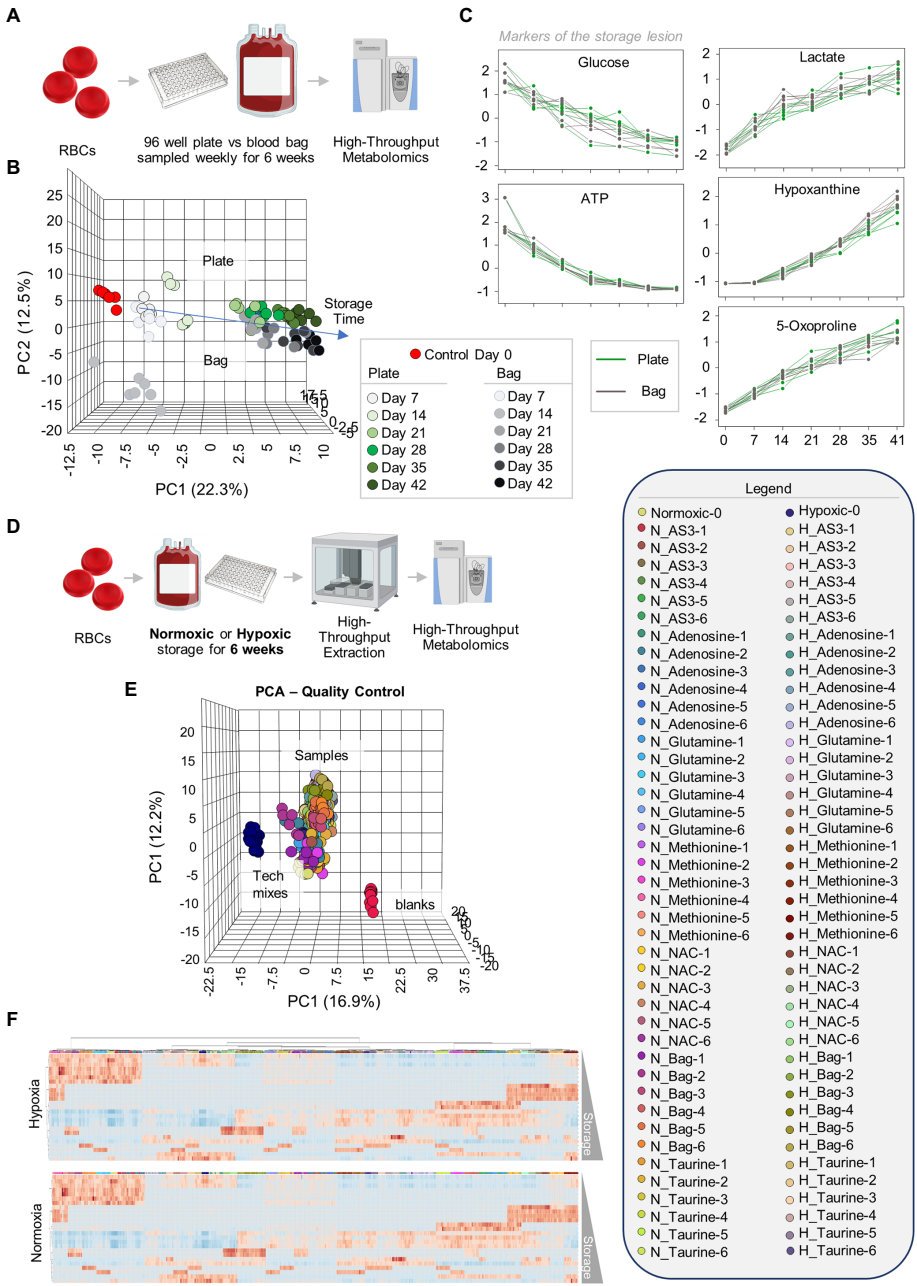
Storage of red blood cells (RBCs) in 96-well plate is comparable to storage in a plastic bag. Leukocyte-filtered RBCs were collected from 12 healthy donors and placed either in a standard pediatric size diethylhexylphtalate (DEHP)-containing bag or in 96-well plate format, prior to storage under refrigerated conditions and weekly sampling for metabolomics **(A)**. **(B)** Similar metabolic phenotypes were observed for RBCs in either storage condition, with overlapping Principal Component Analysis (PCA) traces across component 1 (explaining 22.3% of the total variance) and only minor deviations at storage day 14 across principal component 3 (<5% of the total variance). Samples are color coded with shades of green (plate samples) and gray (bag) from lighter to darker as a function of storage time **(B)**. In **(C)**, line plots show trends for key metabolic markers of the storage lesion as a function of storage duration in the plate (green) or bag (gray). In **(D)**, the experiment in **(A)** was repeated by storing RBCs either in 96 well plates under normoxic (N) or hypoxic (H) conditions, in presence of five different supplements to AS-3 (untreated AS-3, adenosine, glutamine, methionine, N-acetylcysteine—NAC, and Taurine—color-coded in the legend in the right as a function of storage week 1 through 6) or in a small DEHP-supplemented pediatric bags containing AS-3. In **(E)**, PCA shows extreme reproducibility of tech mixes (blue) and blanks (red). In **(F)**, heat maps of hypoxic or normoxic RBCs stored in different additives (significant metabolites by two-way ANOVA are shown).

Leukocyte-filtered RBCs were collected from 12 healthy donor volunteers and added to either standard pediatric size DEHP-containing bags or in 96 well plate format containing AS-3, prior to storage under refrigerated conditions and weekly sampling for metabolomics (Figure 1A). Results are reported in tabulated form in Supplementary Table 1. Similar metabolic phenotypes were observed for RBCs in either storage condition, with overlapping Principal Component Analysis (PCA) traces across component 1 (explaining 22.3% of the total variance) and only minor deviations at storage day 14 across principal component 3 (<5% of the total variance–Figure 1B). Trends for key metabolic markers of the storage lesion as a function of storage duration (Paglia et al., 2016a) followed identical trends over storage in the plate (green) or bag (gray–Figure 1C).

**Figure 2:**
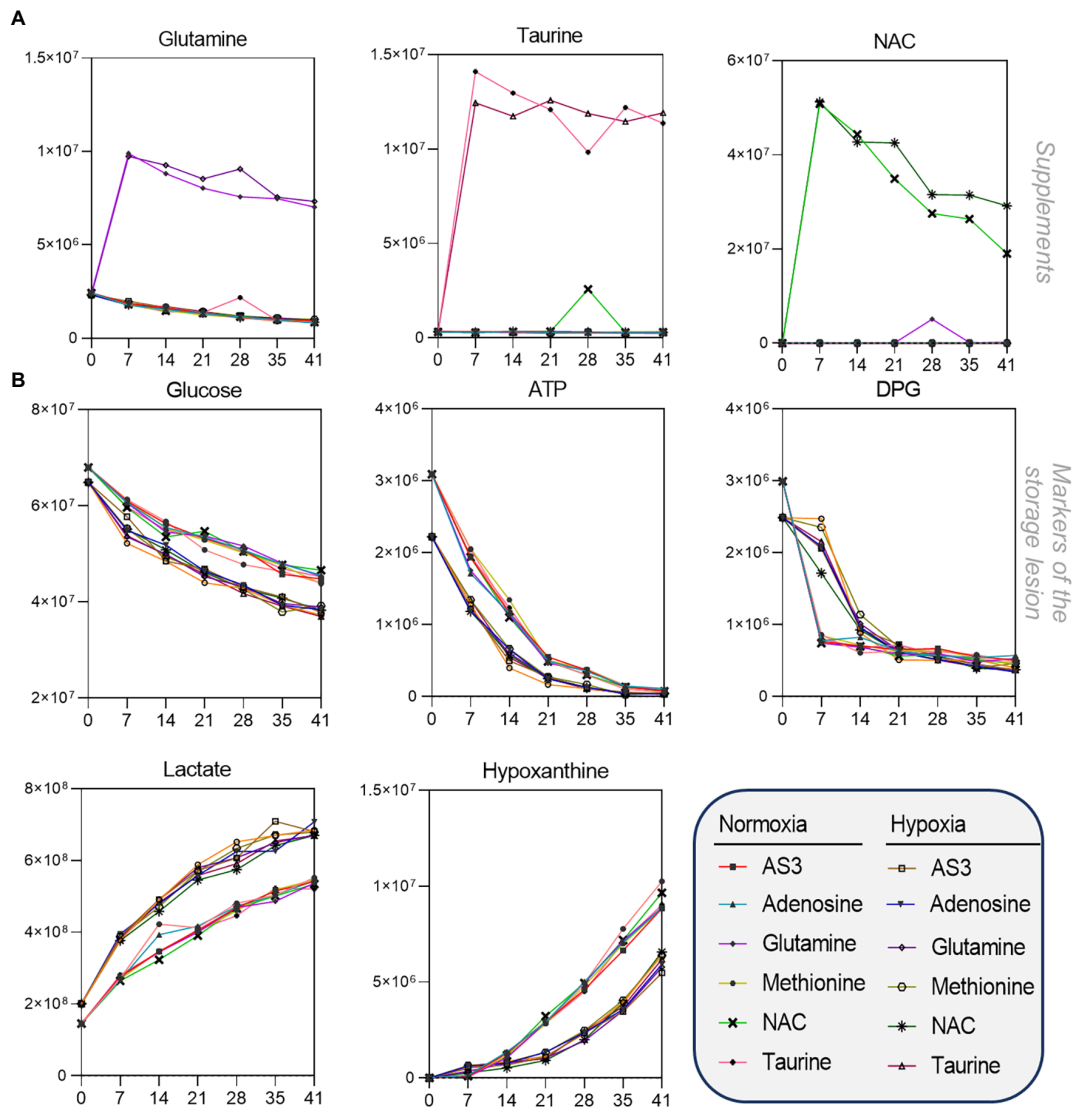
High-throughput metabolomics detects metabolites from the supplements and recapitulates prior studies on metabolic changes during hypoxic storage Line plots in **(A)** highlight glutamine, taurine, and NAC as an example of three metabolites that were supplemented to AS-3, demonstrating that our methods can rapidly detect spiked in substrates and monitor them over time over thousands of samples processed in less than a day. In **(B)**, metabolic markers of the storage lesion (Paglia et al., 2016a; D’alessandro et al., 2017a) show a significant impact of hypoxic storage as a function of storage duration, with identical results to those reported in our previous studies on the impact of hypoxic storage on glycolysis (Reisz et al., 2016) and purine oxidation (e.g., hypoxanthine; Nemkov et al., 2018b). No major impact of any of the experimental supplements on these metabolites was observed.

**Figure 3:**
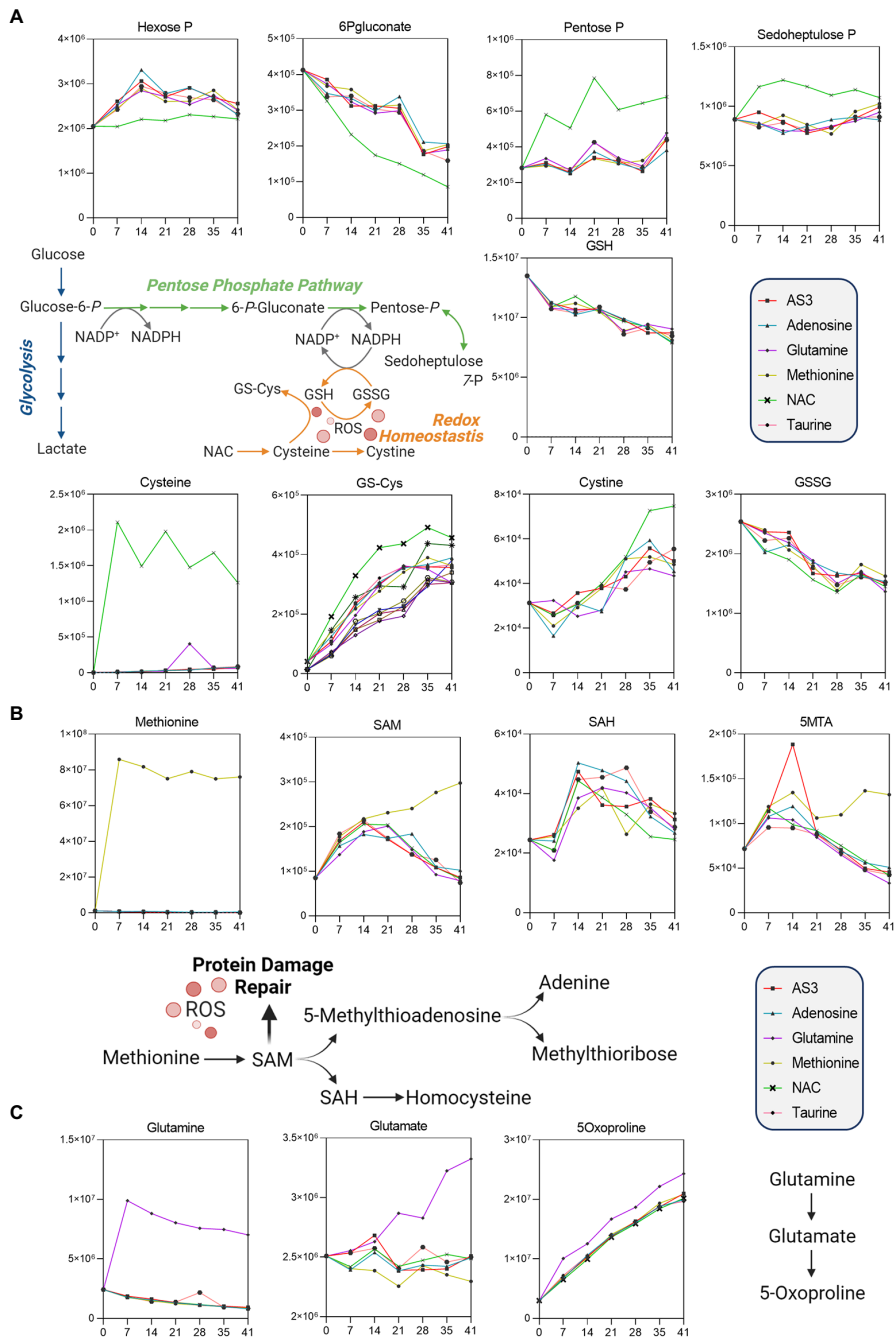
Significant impact of **(A)** NAC on the pentose phosphate pathway, **(B)** methionine on methylation/purine salvage, and **(C)** glutamine on 5-oxoproline levels.

**Figure 4:**
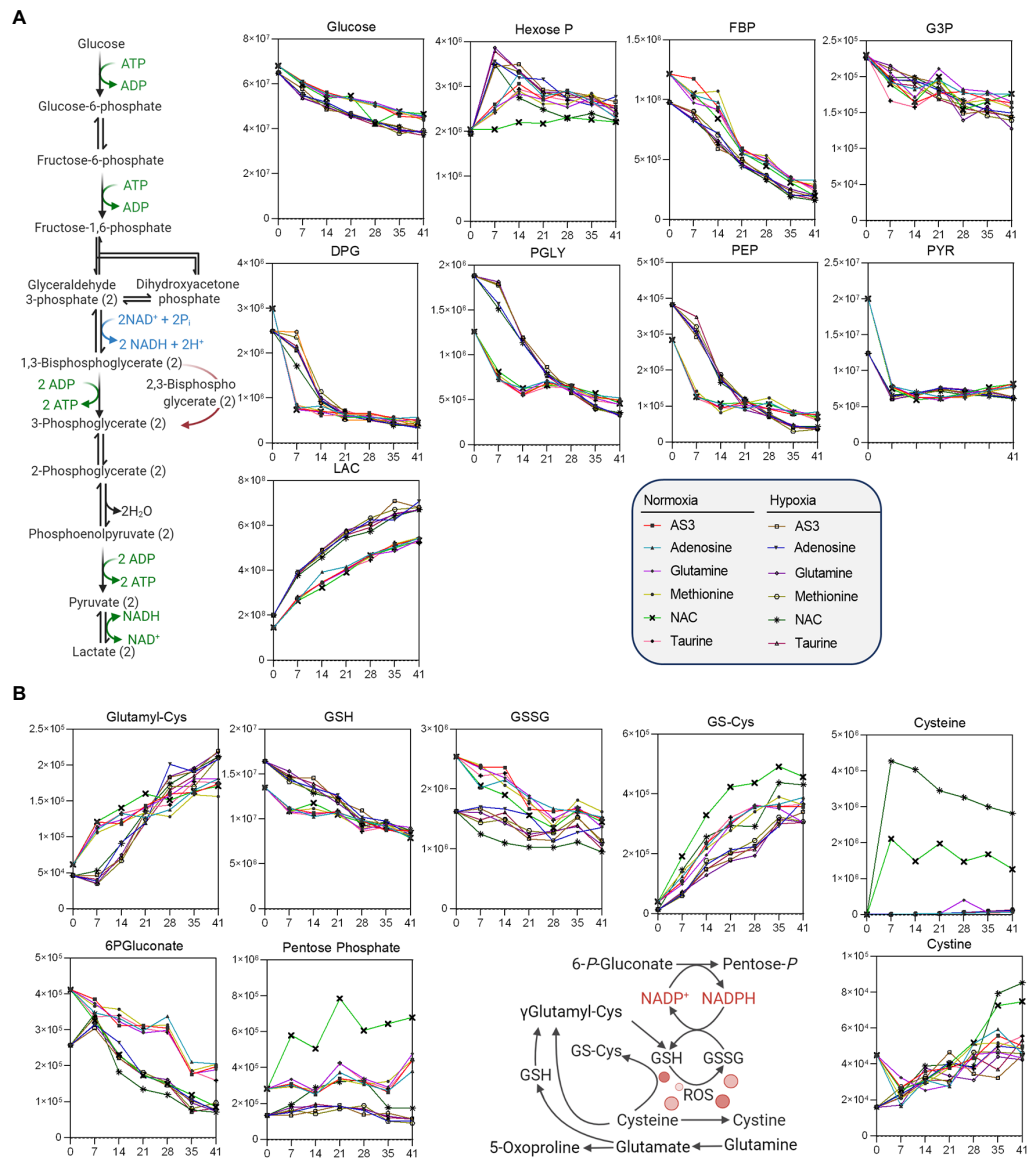
Impact of hypoxia and storage additives on **(A)** glycolysis, **(B)** the pentose phosphate pathway, and **(B)** glutathione homeostasis.

**Figure 5:**
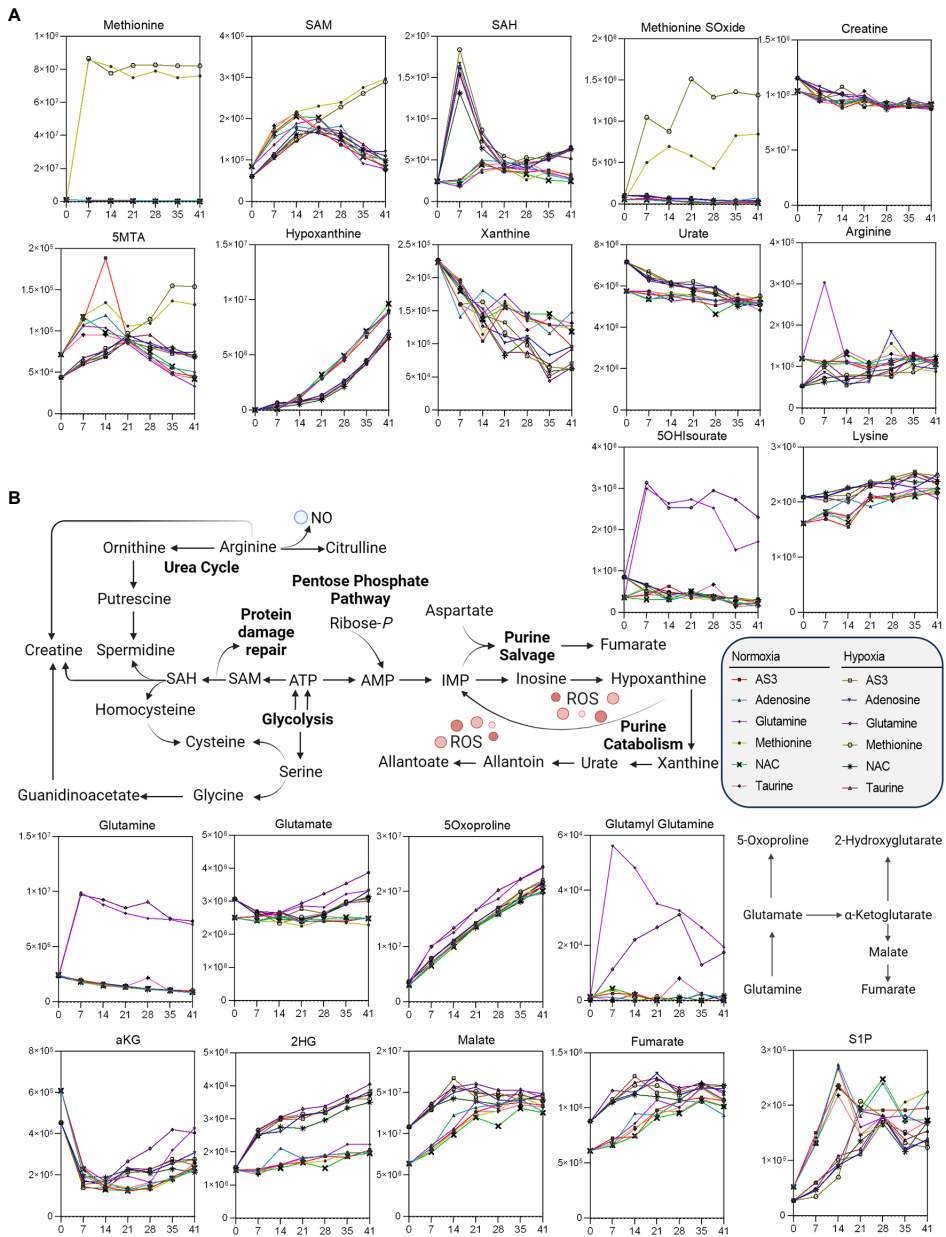
Impact of hypoxia and storage additives on **(A)** methionine metabolism and purine oxidation and salvage, **(B)** glutaminolysis, and carboxylic acid metabolism.

After confirming that storage in 96 well plate format is comparable to storage in the bag, we then set out to determine whether RBCs could be stored at <20% SO_2_ in 96-well plate format. As part of this experiment, we also tested whether the metabolic phenotypes of RBCs stored under hypoxic, refrigerated conditions in 96 well plates would be comparable to the phenotypes of hypoxic RBCs in standard blood bags, as published in previous reports (D’alessandro et al., 2020). Therefore, we repeated the storage experiment described above by storing RBCs in either a standard pediatric size bag or 96-well plate under normoxic or hypoxic conditions. In order to leverage the capacity of the high-throughput screening platform, as an additional variable, we supplemented AS-3 with either adenosine, glutamine, methionine, N-acetylcysteine (NAC), or taurine (Figure 1D). A total of 569 samples, including 30 technical mixes and 18 blanks were processed and run. PCA in Figure 1E shows high technical fidelity tech mixes (a single pooled sample injected repeatedly throughout the entire analysis) and blanks. Significant metabolites were thus determined as a function of storage time and condition (normoxia vs. hypoxia, in presence or absence of supplements to AS-3). Significant metabolites are plotted in the form of a heat map in Figure 1F. Specifically, the platform could reliably quantify the spiked in supplements in normoxic and hypoxic plates (Figure 2 shows examples for glutamine, taurine, and NAC). No significant impact of hypoxia was observed with respect to the metabolism of glutamine or taurine. However, end-of-storage NAC was significantly lower in normoxic RBC (fold change = 0.70, *p* = 0.002) indicating higher consumption to cope with oxidative stress elicited by higher oxygen content. In addition, time course analysis of multiple metabolic markers of the storage lesion (Paglia et al., 2016a) showed a significant impact of hypoxic storage as a function of storage duration (Figure 2B), with identical results to those reported in our previous studies on the impact of hypoxic storage on glycolysis (Reisz et al., 2016) and purine oxidation (hypoxanthine; Nemkov et al., 2018b). No major impact of any of the experimental supplements on these metabolites was observed. Results were further broken down by condition and pathways affected by storage and supplements in Figures 2–5.

### Impact of Supplements to Stored RBC Metabolism Under Normoxic Conditions

Unsupervised analyses highlighted a significant impact of NAC on the pentose phosphate pathway (PPP—Figure 3). Specifically, NAC supplementation corresponded to decreased levels of glucose 6-phosphate (hexose phosphate isomers) substrates of glucose 6-phosphate dehydrogenase, the rate-limiting enzyme of the PPP (Figure 3A). NAC supplementation was accompanied by low levels of 6-phosphogluconate (first intermediate of the oxidative phase of the PPP) and high levels of ribose phosphate (pentose phosphate isobaric isomers) and sedoheptulose phosphate. These observations are suggestive of either an increased activation of the non-oxidative phase of the PPP, or increased fluxes through the oxidative phase—as inferred by law of mass action from the steady state data. However, while NAC supplementation corresponded to higher levels of cysteine, glutathionyl-cysteine, and cystine, no changes in reduced and oxidized glutathione (GSH and GSSG) were noted across the various groups (Figure 3A).

Methionine supplementation was accompanied by increases in the levels of the main methyl-group donor S-adenosylmethionine (SAM), without notable accumulation of its demethylated catabolite S-adenosylhomocysteine (SAH—Figure 3B), though with increases in 5-methylthioadenosine (5MTA).

Glutamine supplementation did not boost glutathione homeostasis, rather fueled synthesis of glutamate and accumulation of its oxidized imino-metabolite, 5-oxoproline (Figure 3C).

### Impact of Hypoxia on Stored RBC Metabolism as a Function of Novel Supplements to AS-3

Additional analyses were performed to further determine whether supplements would differentially fuel RBC metabolism in hypoxia, with a focus on glycolysis, the PPP, and glutathione homeostasis (Figure 4) and methionine metabolism, purine oxidation and salvage, and carboxylic acid metabolism (Figure 5).

As a result, data confirmed a significant impact of hypoxic storage on RBC glycolysis, with increased glucose consumption in hypoxic RBCs, accompanied by lower levels of hexoxe mono- and diphosphate, higher levels of 2,3-diphosphoglycerate (DPG), and downstream triose (phosphates), including phosphoglycerate (PGLY), phosphoenolpyruvate (PEP), and lactate (LAC—Figure 4). Increases in fluxes through glycolysis were accompanied by decreased switch to the pentose phosphate pathway (“normalized” to normoxic AS-3 levels by the supplementation of NAC), with decreased oxidation of cysteine and glutathione in hypoxic RBCs (Figure 4).

Methionine supplementation and consumption were comparable in normoxic and hypoxic RBCs, showing similar trends with respect to the accumulation of SAM as a function of storage. However, lower SAM and higher SAH at storage day 7—as a result of methylation events consuming methyl-groups (D’alessandro et al., 2021b) —was only observed in hypoxic RBCs, which were also characterized by higher levels of methionine S-oxide and 5 methylthioadenosine, as well as decreased markers of purine oxidation (hypoxanthine, xanthine) and higher levels of antioxidant urate (Figure 5). Hypoxia also drove differential lysine and creatine (higher levels than normoxic RBCs) and arginine (lower in hypoxic RBCs than controls—Figure 5), with no significant increases in ornithine, citrulline, or polyamines (not shown).

Glutaminolysis and hypoxia were the main driver of accumulation of carboxylic acids, such as the product of glutamate-dependent transamination, alpha-ketoglutarate (aKG), and downstream metabolites 2-hydroxyglutarate (2HG), malate, and fumarate (Figure 5), with no detectable changes in the levels of succinate (not shown). Finally, storage induced the accumulation of S1P in both normoxic and hypoxic RBCs (Figure 5).

### Impact of Hypoxia on Glycolysis to Pentose Phosphate Pathway Flux

**Figure 6:**
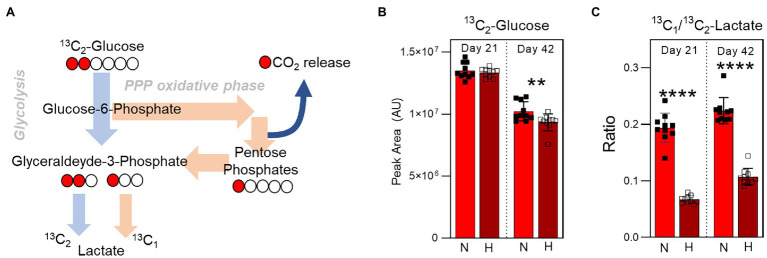
Isotope tracing analysis to disentangle glycolysis vs. pentose phosphate pathway utilization. **(A)** The labeling scheme of ^13^C_1,2_-Glucose is shown, which depicts the relative contribution of ^13^C_2_-lactate and ^13^C_1_-lactate to the total lactate pool by glycolysis and the pentose phosphate pathway, respectively. **(B)** The relative levels (in peak area, arbitrary units) of ^13^C_2_-Glucose at Day 21 (left) and Day 42 (right) in RBC stored under normoxic (light red) or hypoxic (dark red) conditions. **(C)** The ratio of ^13^C_1_-lactate to ^13^C_2_-lactate in the same samples. *p*-values from a two-tailed paired T-test comparing normoxic and hypoxic storage at each time point are indicated as **p < 0.01; and ****p < 0.0001.

To determine the relative utilization of glycolysis and the pentose phosphate pathway as a function of both storage duration and oxygen levels, 1,2-^13^C_2_-glucose was spiked into AS-3 at the beginning of the storage period. The presence of ^13^C enables the determination of kinetics and amount of lactate production during storage. Specifically, glucose that is processed through glycolysis for lactate generation results in the production of ^13^C_2_-lactate. However, any glucose that is re-routed through the pentose phosphate pathway and back into glycolysis through the activities of transketolase and transaldolase results in the production of ^13^C_1_-lactate, as the other ^13^C is lost as CO_2_ during the conversion of 6-phosphogluconate to ribulose-5-phosphate by 6-phosphogluconate dehydrogenase (Figure 6A). Therefore, comparing the relative amounts of ^13^C_1_- and ^13^C_2_-lactate enables monitoring of pathway flux. While the amount of ^13^C_2_-glucose was comparable in normoxia and hypoxia 3 weeks into storage, end of storage glucose was lower in the hypoxic samples indicating higher utilization (Figure 6B). Meanwhile, ^13^C_1_/^13^C_2_-lactate ratio increased in both oxygen conditions between 3 and 6 weeks of storage, but was significantly higher in the normoxic samples at both time points (Figure 6C). These results indicate higher pentose phosphate pathway utilization in the normoxic stored samples, supporting previous steady state results (Figure 4).

## Discussion

In the present study, we describe a novel high-throughput metabolomics platform for the storage and rapid screening of novel additives for RBC storage under normoxic or hypoxic/hypocapnic conditions. To show feasibility of the approach, we compared the phenotypes of paired RBCs stored either in 96-well plate format, standard, or paediatric-sized bags until the end of their shelf life of 42 days. Results confirmed that the 96-well plate platform is comparable to storage in large units, while allowing the simultaneous testing of multiple storage additives/supplements and conditions (herein, normoxia, and hypoxia). Trends for key metabolic markers of the storage lesion as a function of storage duration (Paglia et al., 2016a) followed identical trends to those reported before for RBCs stored in AS-3, under normoxic or hypoxic conditions (D’alessandro et al., 2020). Storage conditions and supplements to AS-3 were purposely selected here to mimic multiple studies in the literature where novel formulations were tested under normoxic conditions, to determine whether our platforms could recapitulate decades of studies in a matter of a single day of mass spectrometry run, as a form of internal validation. The novelty of the study relies, however, not only just on the method, but also on the testing of the very same additives and supplements within the framework of blood storage under hypoxic conditions in a scalable, high-throughput format. The method allows to significantly expedite and multiplex sample extraction in 96-well plate format (allowing us to prepare up to 5,000 samples/day compared to previous ~500/day), as well as run time on the instrument (~5x faster than our previous methods; Nemkov et al., 2019). These numbers also translate in critical decreases in the processing costs, in terms of instrument time (5-fold lower $/sample), with comparable costs for consumables.

In prior studies, glutamine supplementation (either unlabeled or ^13^C ^15^N-glutamine) had been proposed as a strategy to boost glutathione synthesis by feeding glutamine-derived glutamate generation (Whillier et al., 2011; D’alessandro et al., 2017b). Both prior studies concluded on the lack of efficacy of this strategy, perhaps as a function of decreasing ATP in stored RBCs (Xiong et al., 2018) since *de novo* glutathione synthesis is an ATP-dependent process. Indeed, hypoxic and hypocapnic storage of RBC was found to prevent ATP consumption in stored units, while fueling GSH synthesis (Yoshida et al., 2017). As such, here we tested whether glutamine could boost glutathione production in hypoxic, hypocapnic RBCs during storage. However, results did not show a specific benefit of glutamine supplementation in promoting GSH synthesis beyond the basal levels from all the other supplements tested herein.

Previous studies had suggested the use of the antioxidant NAC as a strategy to mitigate storage-induced oxidant stress (Pallotta et al., 2014; Bayer et al., 2015; Amen et al., 2017). Our platform recapitulates these results and expands on them by providing a comprehensive overview of the beneficial impact of NAC supplementation on PPP activation and the preservation of free thiols (cysteine, glutathionyl-cysteine).

Similarly, previous studies had shown that methionine consumption is significantly elevated in stored RBCs from patients with high oxidative hemolysis, i.e., the RBC susceptibility to hemolyze following oxidant insults (Reisz et al., 2018). Methionine was indeed found to feed oxidant stress-induced isoaspartyl-protein damage repair through the activity of the enzyme PIMT. Genetic ablation of PIMT does result in RBCs that are more susceptible to intra- and extra-vascular hemolysis following oxidant stress *in vitro* and *in vivo* (D’alessandro et al., 2021b). Supplementation of methionine to the additive solution would thus not only provide a direct scavenger of ROS, but also fuel repairing of oxidatively damaged RBCs. Notably, methionine supplementation was associated with increased availability of the main donor/reservoir of methyl-groups (SAM). Of note, a previous study from our group found that the ratio of SAM-to-SAH was higher toward the end of storage under hypoxic conditions (Reisz et al., 2018). Since difference in methyl consumption for protein damage repair may be donor dependent (D’alessandro et al., 2021a) on the basis of genetic and environmental factors, the ability to screen multiple samples in parallel using the 96-well-plate storage platform described here offers the potential to assess donor-dependent responses to a variety of storage supplements, thereby helping to realize the potential of personalized transfusion medicine (D’alessandro and Liumbruno, 2019). Despite altered SAM/SAH ratios, however, storage-induced increases in 5-methylthioadenosine (5MTA)—especially in normoxic, methionine-supplemented RBCs. These results are consistent with previous studies on the activation of purine salvage reactions in stored RBCs as a function of storage-induced oxidant stress in AS-3 and SAGM (Paglia et al., 2016b). Notably, slower increases in purine oxidation (hypoxanthine, xanthine) and preservation of the antioxidant urate (Tzounakas et al., 2018) had already been described in RBCs undergoing hypoxic storage (D’alessandro et al., 2020), further confirming the comparability of the new platform to storage in the plastic bag.

Another sulfur-containing antioxidant compound, taurine was previously reported to boost RBC energy and redox metabolism, as well as post-transfusion recoveries in murine models of blood storage (Bertolone et al., 2020). However, the effect of taurine on normoxic RBCs was here found to be negligible compared to the changes imparted by hypoxic storage and/or other additive supplements, highlighting the importance of being able to test multiple conditions in parallel on the very same samples.

Increases in the levels of several carboxylates, including fumarate and malate, as well as glutamine-derived aKG, 2HG, but not succinate—as expected in mitochondria-devoid RBCs—is consistent with previous findings on carboxylate metabolism in RBCs as a function of oxygen levels (Nemkov et al., 2017b).

S1P increases were observed as a function of storage in normoxic and hypoxic samples, though—unexpectedly (Sun et al., 2016) —at a lower rate in the latter group. Previous studies have shown that S1P levels intracellularly are regulated by the Mfsd2b transporter (Kurano et al., 2017), whose polymorphisms in the blood donor population associate with which increased susceptibility to osmotic stress (Kurano et al., 2017). Mfsd2b responds to proton gradients and band 3 activity as an anion exchanger in the chloride shift. As such, one can speculate that S1P accumulation may be due to a combined effect of hypoxia-induced increase in intracellular acidification (D’alessandro et al., 2020) and increased protection of band 3-damage (Issaian et al., 2021). Finally, relevant to band 3 role in the regulation of RBC metabolic responses to hypoxia, the oxygen-dependent metabolic switch between glycolysis and the pentose phosphate pathway (Issaian et al., 2021; Rogers et al., 2021) is indeed preserved in this platform, as evidenced by intracellular isotopic labeling studies with ^13^C-glucose.

The present study holds several limitations. A limited number of additives, some of which previously described in the literature, were tested in this study. The rationale behind this choice is explained by the proof-of-feasibility nature of the present study. Indeed, here, we demonstrate the feasibility of blood storage (in normoxic and hypoxic conditions) in a 96-well-plate format. We then performed a multiplexed testing of storage conditions *via* high-throughput metabolomics screening, and documented that the results obtained with the new platform are comparable to the existing literature. Follow up studies are currently underway to determine the impact of hundreds of additional supplements (or mixes of, as explored for a subset of additives by others in prior work; Whillier et al., 2011), across different ranges of concentration, over multiple pH ranges.

Metabolomics can provide a high-throughput cost-effective readout of the energy and redox status of the RBCs. Biomarkers of functional readouts, such as O_2_ kinetics (Donovan et al., 2021), hemolysis (D’alessandro et al., 2021a), and post-transfusion recovery (D’alessandro et al., 2020) have been described. However, follow-up analyses for selected additives should directly assess such functional measurements, including determination of DPG levels and hemoglobin oxidation status. To this end, it is worth noting that our hemoglobin autoxidation has been reported in cell free conditions under hypoxia (Sadrzadeh et al., 1984). Superoxide production during the autoxidation of hemoglobin is facilitated under hypoxic conditions where hemoglobin is only partially oxygenated, though the maximum rate of superoxide production is observed in the region of 25 mm Hg (Rifkind et al., 1991). On the other hand, at much lower pressures, where the hemoglobin is mostly deoxygenated, the rate of lysis is dramatically decreased with almost no lysis detected even after 3 days (Rifkind et al., 1991). In this view, it is worth noting that the hypoxic conditions described here are ~15 mmHg (highest value, range 5–15 mmHg), well below the 25 mmHg pO2 range for superoxide formation. In addition, autoxidation is most pronounced for free hemoglobin (e.g., following cell lysis) under hypoxic conditions in the microcirculation and for unstable dimers formed at reduced hemoglobin concentrations. As Rifkind noted, in the RBC oxidative reactions are inhibited by an extensive antioxidant system (Rifkind et al., 2015). This is in keeping with prior work showing a lower rate of hemoglobin oxidation (e.g., irreversible C93 oxidation) under hypoxic storage conditions (Reisz et al., 2016), has documented to be better preserved under hypoxic storage conditions. In this view, the observations in the 96-well plate format recapitulate prior data, showing an enhanced energy metabolism and mitigated oxidant stress in hypoxic RBCs. Such a metabolic advantage of hypoxic storage over standard storage conditions had been previously shown to correspond to a higher post-transfusion recovery in recent clinical trials (D’alessandro et al., 2020).

In conclusion, here we described a novel high-throughput RBC storage platform for the rapid metabolomics testing of multiple conditions, including hypoxic storage and several additives. We validated this platform against well-established literature on a handful of supplements to stored RBCs and tested whether any of those would improve the quality of hypoxically stored RBCs. While merely a proof of principle, this study paves the way for the multiplexed, high-throughput data-driven design and testing of novel formulation for RBC storage in control or hypoxic conditions.

## Data Availability Statement

The original contributions presented in the study are included in the article/Supplementary Material, further inquiries can be directed to the corresponding authors.

## Ethics Statement

Ethical review and approval was not required for the study on human participants in accordance with the local legislation and institutional requirements. The patients/participants provided their written informed consent to participate in this study.

## Author Contributions

TN, TY, and AD designed the study. MN and TY performed storage studies. TN and AD performed metabolomics analyses (untargeted and targeted quantitative), tracing experiments, and data analysis and prepared the figures and tables. AD wrote and modified the first draft of the manuscript, which was revised by all the other authors. All authors contributed to the article and approved the submitted version.

## Funding

Research reported in this publication was supported by funds from R44HL149579 and R44GM130268 (TY and TN) by the National Heart, Lung and Blood Institutes. Additional funds received by the authors are acknowledged, including R01HL146442, R01HL149714, R01HL148151, R01HL161004, and R21HL150032 by the National Heart, Lung and Blood Institutes (AD) and RM1GM131968 by the National Institute of General and Medical Sciences (AD).

## Conflict of Interest

AD and TN are founders of Omix Technologies Inc. and Altis Biosciences LLC. AD is an advisory board member of Hemanext Inc. and Forma Therapeutics Inc. TY and MN are employees of Hemanext Inc.

## Publisher’s Note

All claims expressed in this article are solely those of the authors and do not necessarily represent those of their affiliated organizations, or those of the publisher, the editors and the reviewers. Any product that may be evaluated in this article, or claim that may be made by its manufacturer, is not guaranteed or endorsed by the publisher.

## Footnotes

^1^ www.hmdb.ca

## Supplementary Material

Supplementary Figure 1 Line plots show the impact of additives and storage on RBC metabolites under control or hypoxic conditions.
