# Supplementary figures and images for: High-Throughput Metabolomics Platform for the Rapid Data-Driven Development of Novel Additive Solutions for Blood Storage
Source: Front Physiol. 2022 Mar 14;13:833242. doi: 10.3389/fphys.2022.833242 (PMC8964052; doi:10.3389/fphys.2022.833242)

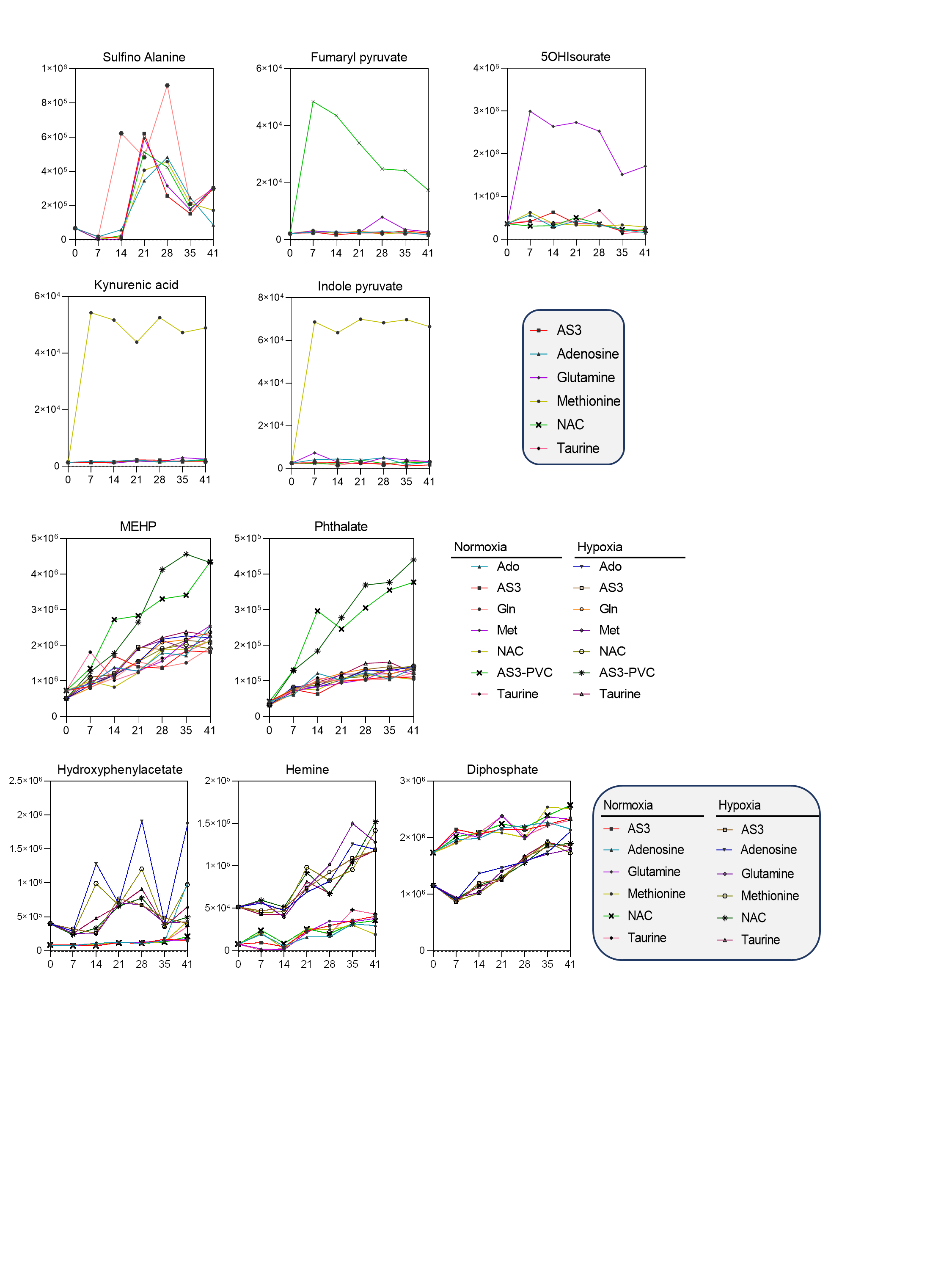

Supplement: Supplementary Figure 1 — Line plots show the impact of additives and storage on RBC metabolites under control or hypoxic conditions. [file Image_1.TIF]
